# Tetra­kis(μ-2-phen­oxy­propionato)-κ^3^ *O*,*O*′:*O*′;κ^3^ *O*:*O*,*O*′,κ^4^ *O*:*O*′-bis­[(1,10-phenanthroline-κ^2^ *N*,*N*′)(2-phen­oxy­propionato-κ^2^ *O*,*O*′)praseodymium(III)]

**DOI:** 10.1107/S1600536811034702

**Published:** 2011-08-31

**Authors:** Jin-Bei Shen, Jia-Lu Liu, Guo-Liang Zhao

**Affiliations:** a College of Chemistry and Life Sciences, Zhejiang Normal University, Jinhua 321004, Zhejiang, People’s Republic of China; b Zhejiang Normal University Xingzhi College, Jinhua, Zhejiang 321004, People’s Republic of China

## Abstract

In the centrosymmetric binuclear title complex, [Pr_2_(C_9_H_9_O_3_)_6_(C_12_H_8_N_2_)_2_], the two Pr^III^ ions are linked by four 2-phen­oxy­propionate (*L*) groups through their bi- and tridentate bridging modes. Each Pr^III^ ion is nine-coordinated by one 1,10-phenanthroline mol­ecule, one bidentate carboxyl­ate group and four bridging carboxyl­ate groups in a distorted PrN_2_O_7_ monocapped square-anti­prismatic geometry. The title compound is isotypic with its terbium- and dysprosium-containing analogues.

## Related literature

For the isotypic Tb and Dy compounds, see: Shen *et al.* (2011*a* ▶,*b* ▶). For a related structure, see: Li *et al.* (2008 ▶).
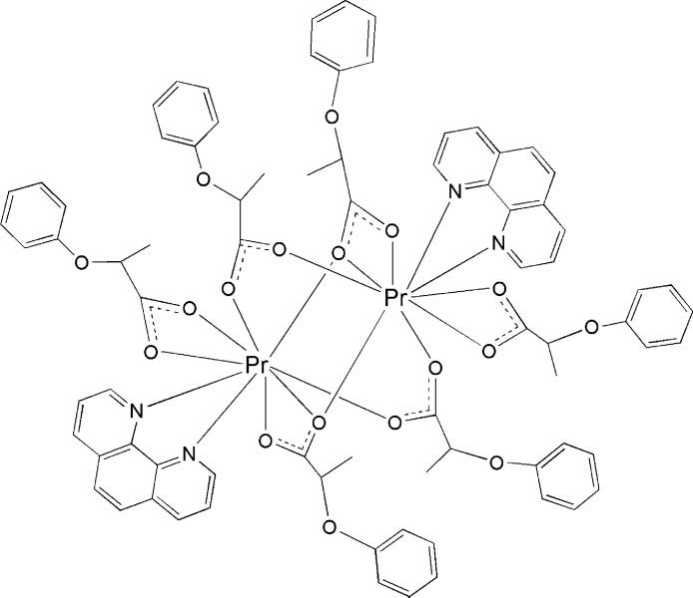


## Experimental

### 

#### Crystal data

[Pr_2_(C_9_H_9_O_3_)_6_(C_12_H_8_N_2_)_2_]
*M* *_r_* = 1633.20
Monoclinic, 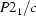

*a* = 11.5142 (2) Å
*b* = 25.8845 (4) Å
*c* = 13.9275 (2) Å
β = 120.204 (1)°
*V* = 3587.41 (10) Å^3^
*Z* = 2
Mo *K*α radiation
μ = 1.42 mm^−1^
*T* = 296 K
0.39 × 0.15 × 0.11 mm

#### Data collection

Bruker APEXII CCD diffractometer
Absorption correction: multi-scan (*SADABS*; Sheldrick, 1996 ▶) *T*_min_ = 0.779, *T*_max_ = 0.862
48041 measured reflections
6322 independent reflections
5424 reflections with *I* > 2σ(*I*)
*R*_int_ = 0.033

#### Refinement

*R*[*F* ^2^ > 2σ(*F* ^2^)] = 0.022
*wR*(*F* ^2^) = 0.051
*S* = 1.02
6322 reflections
464 parameters
H-atom parameters constrained
Δρ_max_ = 0.36 e Å^−3^
Δρ_min_ = −0.30 e Å^−3^

### 

Data collection: *APEX2* (Bruker, 2006 ▶); cell refinement: *SAINT* (Bruker, 2006 ▶); data reduction: *SAINT*; program(s) used to solve structure: *SHELXS97* (Sheldrick, 2008 ▶); program(s) used to refine structure: *SHELXL97* (Sheldrick, 2008 ▶); molecular graphics: *SHELXTL* (Sheldrick, 2008 ▶); software used to prepare material for publication: *SHELXL97*.

## Supplementary Material

Crystal structure: contains datablock(s) I, global. DOI: 10.1107/S1600536811034702/hb6378sup1.cif

Structure factors: contains datablock(s) I. DOI: 10.1107/S1600536811034702/hb6378Isup2.hkl

Additional supplementary materials:  crystallographic information; 3D view; checkCIF report

## Figures and Tables

**Table 1:** Selected bond lengths (Å)

Pr1—O5^i^	2.4215 (15)
Pr1—O7	2.4320 (15)
Pr1—O8^i^	2.4657 (15)
Pr1—O2	2.5117 (17)
Pr1—O1	2.5324 (16)
Pr1—O4	2.5501 (15)
Pr1—N1	2.6199 (18)
Pr1—O5	2.6755 (15)
Pr1—N2	2.6782 (18)

Symmetry code: (i) 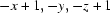.

**Fig. 1.**
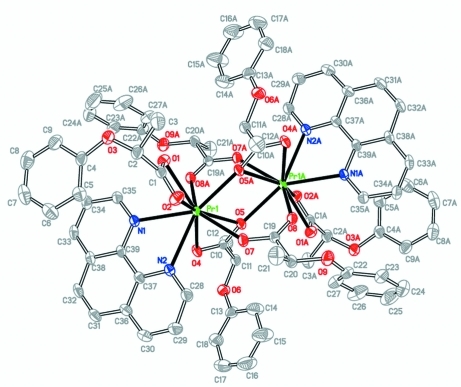
The molecular structure of (I) with displacement ellipsoids drawn at the 30% probability level.

**Fig. 2.**
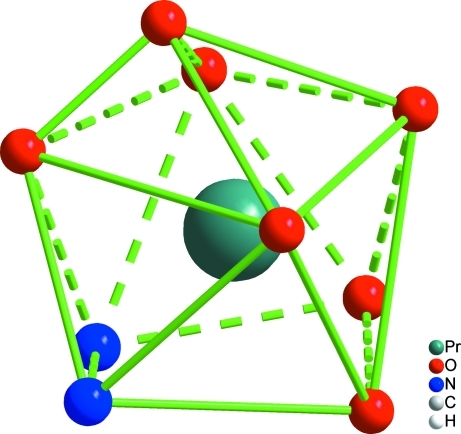
The coordination environment of the Pr(III) ion.

## supplementary crystallographic information

### Comment

As part of our ongoing studies of 2-phenoxypropionic acid complexes (Shen *et al.*, 2011*a*,*b*) we now describe the title Pr^III^ complex.

The structure of the title compound (1) is a dinuclear praseodymium complex with Pr—PrA separation of 4.0785 (2) Å. The structure of the complex (Fig. 1) reveals that the molecule contains six *L*, two phen molecules and two Pr^III^ ions. Each Pr(III) ion is coordinated to nine atoms, of which five oxygen atoms are from the bridging carboxylates, two oxygen atoms from the bidentate chelating carboxylate group, and two nitrogen atoms from a 1,10- phenanthroline molecule. The *L* ligands are coordinated to the Pr^III^ ions in three different modes: chelating,bridging and bridging tridentate.The analysis of structural features indicates that the central Pr(III) ion adopts a distorted monocapped square antiprism geometry(Fig. 2).The Pr—O distances are all within the range 2.4215 (15)–2.6755 (15) Å, and the Pr—N distances rang from 2.6199 (18)–2.6782 (18) Å, all of which are within the range of those of other nine-coordinated Pr^III^ complexes with carboxylic donor ligands and 1,10-phenanthroline (Li *et al.*, 2008). The selected bond lengths and angles for complex 1 are listed in Table 1. In addition, there are no classical hydrogen bonds in the crystal structure, because good hydrogen bond donors are absent. The most significant intermolecular interactions are C—H···O hydrogen bonds (Table 2) and weak π···π aromatic interactions from phen molecules and aromatic rings of the *L* ligands.

### Experimental

Reagents and solvents used were of commercially available quality and without purified before using. 2-phenoxypropionic acid (1.5 mmol), Pr(NO_3_)_3_.6H_2_O (0.5 mmol) and 1,10-phenanthroline (0.5 mmol) were dissolved in 20 ml e nthanol, then 10 ml water was added to the above solution. The mixed solution was stirred for 12 h at room temperature. At last, deposit was filtered out and the colourless solution was kept in the open air. The colourless crystal was obtained after several days.

### Refinement

The structure was solved by direct methods and successive Fourier difference synthesis. The H atoms bonded to C and N atoms were positioned geometrically and refined using a riding model [aliphatic C—H =0.96 Å (*U*_iso_(H) = 1.5*U*_eq_(C)), aromatic C—H = 0.93 Å (*U*_iso_(H) = 1.2*U*_eq_(C))].

### Crystal data

**Table:**

[Pr_2_(C_9_H_9_O_3_)_6_(C_12_H_8_N_2_)_2_]	*F*(000) = 1656
*M**_r_* = 1633.20	*D*_x_ = 1.512 Mg m^−^^3^
Monoclinic, *P*2_1_/*c*	Mo *K*α radiation, λ = 0.71073 Å
Hall symbol: -P 2ybc	Cell parameters from 9918 reflections
*a* = 11.5142 (2) Å	θ = 1.9–25.0°
*b* = 25.8845 (4) Å	µ = 1.42 mm^−^^1^
*c* = 13.9275 (2) Å	*T* = 296 K
β = 120.204 (1)°	Block, colourless
*V* = 3587.41 (10) Å^3^	0.39 × 0.15 × 0.11 mm
*Z* = 2	

### Data collection

**Table:**

Bruker APEXII CCD diffractometer	6322 independent reflections
Radiation source: fine-focus sealed tube	5424 reflections with *I* > 2σ(*I*)
graphite	*R*_int_ = 0.033
φ and ω scans	θ_max_ = 25.0°, θ_min_ = 1.9°
Absorption correction: multi-scan (*SADABS*; Sheldrick, 1996)	*h* = −13→13
*T*_min_ = 0.779, *T*_max_ = 0.862	*k* = −30→30
48041 measured reflections	*l* = −16→16

### Refinement

**Table:**

Refinement on *F*^2^	Primary atom site location: structure-invariant direct methods
Least-squares matrix: full	Secondary atom site location: difference Fourier map
*R*[*F*^2^ > 2σ(*F*^2^)] = 0.022	Hydrogen site location: inferred from neighbouring sites
*wR*(*F*^2^) = 0.051	H-atom parameters constrained
*S* = 1.02	*w* = 1/[σ^2^(*F*_o_^2^) + (0.0206*P*)^2^ + 2.173*P*] where *P* = (*F*_o_^2^ + 2*F*_c_^2^)/3
6322 reflections	(Δ/σ)_max_ = 0.001
464 parameters	Δρ_max_ = 0.36 e Å^−^^3^
0 restraints	Δρ_min_ = −0.30 e Å^−^^3^

### Special details

**Table:**

Geometry. All e.s.d.'s (except the e.s.d. in the dihedral angle between two l.s. planes) are estimated using the full covariance matrix. The cell e.s.d.'s are taken into account individually in the estimation of e.s.d.'s in distances, angles and torsion angles; correlations between e.s.d.'s in cell parameters are only used when they are defined by crystal symmetry. An approximate (isotropic) treatment of cell e.s.d.'s is used for estimating e.s.d.'s involving l.s. planes.
Refinement. Refinement of *F*^2^ against ALL reflections. The weighted *R*-factor *wR* and goodness of fit *S* are based on *F*^2^, conventional *R*-factors *R* are based on *F*, with *F* set to zero for negative *F*^2^. The threshold expression of *F*^2^ > σ(*F*^2^) is used only for calculating *R*-factors(gt) *etc*. and is not relevant to the choice of reflections for refinement. *R*-factors based on *F*^2^ are statistically about twice as large as those based on *F*, and *R*- factors based on ALL data will be even larger.

### Fractional atomic coordinates and isotropic or equivalent isotropic displacement parameters (Å^2^)

**Table:**

	*x*	*y*	*z*	*U*_iso_*/*U*_eq_	
Pr1	0.546191 (11)	0.002649 (4)	0.661778 (9)	0.02753 (5)	
O1	0.46480 (16)	0.08618 (6)	0.70264 (14)	0.0424 (4)	
O2	0.37328 (19)	0.01491 (7)	0.71925 (16)	0.0492 (4)	
O3	0.4013 (2)	0.13518 (7)	0.84788 (15)	0.0565 (5)	
O4	0.76114 (15)	−0.04330 (6)	0.69975 (12)	0.0377 (4)	
O5	0.62273 (15)	−0.03201 (6)	0.52110 (12)	0.0369 (4)	
O6	0.91161 (16)	−0.11113 (6)	0.65422 (15)	0.0463 (4)	
O7	0.41578 (17)	−0.07295 (6)	0.56205 (13)	0.0426 (4)	
O9	0.17325 (18)	−0.15454 (7)	0.34639 (15)	0.0534 (5)	
N1	0.72109 (19)	0.03022 (7)	0.86545 (15)	0.0347 (4)	
N2	0.62664 (19)	−0.06860 (7)	0.82227 (15)	0.0347 (4)	
C1	0.3932 (2)	0.06264 (10)	0.73207 (19)	0.0382 (6)	
C2	0.3224 (3)	0.09314 (10)	0.7824 (2)	0.0487 (7)	
H2	0.3025	0.0701	0.8280	0.058*	
C3	0.1929 (3)	0.11599 (13)	0.6903 (3)	0.0710 (10)	
H3A	0.1486	0.1348	0.7221	0.106*	
H3B	0.1353	0.0887	0.6443	0.106*	
H3C	0.2127	0.1389	0.6462	0.106*	
C4	0.5091 (3)	0.12558 (10)	0.9521 (2)	0.0467 (6)	
C5	0.5450 (3)	0.07740 (11)	1.0007 (2)	0.0537 (7)	
H5	0.4984	0.0481	0.9618	0.064*	
C6	0.6518 (3)	0.07361 (14)	1.1085 (3)	0.0663 (9)	
H6	0.6765	0.0414	1.1424	0.080*	
C7	0.7217 (3)	0.11654 (16)	1.1663 (3)	0.0721 (10)	
H7	0.7927	0.1136	1.2389	0.087*	
C8	0.6857 (3)	0.16404 (15)	1.1155 (3)	0.0723 (9)	
H8	0.7334	0.1933	1.1537	0.087*	
C9	0.5805 (3)	0.16858 (12)	1.0096 (3)	0.0614 (8)	
H9	0.5570	0.2009	0.9760	0.074*	
C10	0.7320 (2)	−0.04845 (8)	0.60161 (18)	0.0298 (5)	
C11	0.8302 (2)	−0.07360 (9)	0.5733 (2)	0.0385 (5)	
H11	0.7804	−0.0901	0.5002	0.046*	
C12	0.9243 (3)	−0.03343 (11)	0.5717 (3)	0.0565 (7)	
H12A	0.9854	−0.0496	0.5530	0.085*	
H12B	0.9742	−0.0177	0.6437	0.085*	
H12C	0.8732	−0.0075	0.5174	0.085*	
C13	0.8491 (3)	−0.15404 (10)	0.6641 (2)	0.0493 (7)	
C14	0.7182 (3)	−0.16731 (12)	0.5909 (3)	0.0878 (12)	
H14	0.6647	−0.1461	0.5306	0.105*	
C15	0.6667 (4)	−0.21245 (14)	0.6076 (4)	0.1154 (17)	
H15	0.5785	−0.2217	0.5570	0.139*	
C16	0.7412 (4)	−0.24346 (14)	0.6956 (4)	0.0997 (14)	
H16	0.7047	−0.2735	0.7060	0.120*	
C17	0.8718 (4)	−0.22981 (13)	0.7696 (3)	0.0886 (12)	
H17	0.9243	−0.2507	0.8307	0.106*	
C18	0.9250 (3)	−0.18527 (12)	0.7534 (3)	0.0687 (9)	
H18	1.0135	−0.1763	0.8038	0.082*	
C19	0.3420 (2)	−0.08871 (8)	0.46418 (19)	0.0341 (5)	
C20	0.2600 (2)	−0.13672 (9)	0.4556 (2)	0.0417 (6)	
H20	0.3228	−0.1646	0.4975	0.050*	
C21	0.1745 (3)	−0.12638 (12)	0.5076 (3)	0.0660 (9)	
H21A	0.1244	−0.1569	0.5027	0.099*	
H21B	0.1136	−0.0985	0.4689	0.099*	
H21C	0.2313	−0.1171	0.5842	0.099*	
C22	0.2245 (3)	−0.18306 (9)	0.2932 (2)	0.0501 (7)	
C23	0.1281 (4)	−0.20964 (11)	0.2017 (3)	0.0738 (10)	
H23	0.0385	−0.2087	0.1832	0.089*	
C24	0.1666 (6)	−0.23746 (15)	0.1385 (3)	0.1089 (16)	
H24	0.1024	−0.2555	0.0767	0.131*	
C25	0.2973 (7)	−0.23900 (16)	0.1650 (4)	0.1140 (18)	
H25	0.3223	−0.2582	0.1218	0.137*	
C26	0.3914 (5)	−0.21248 (13)	0.2546 (4)	0.0915 (12)	
H26	0.4806	−0.2133	0.2720	0.110*	
C27	0.3564 (3)	−0.18453 (10)	0.3201 (3)	0.0627 (8)	
H27	0.4215	−0.1668	0.3819	0.075*	
C28	0.5822 (3)	−0.11652 (10)	0.8031 (2)	0.0465 (6)	
H28	0.5085	−0.1242	0.7340	0.056*	
C29	0.6391 (3)	−0.15632 (10)	0.8804 (2)	0.0567 (7)	
H29	0.6030	−0.1895	0.8633	0.068*	
C30	0.7479 (3)	−0.14596 (11)	0.9811 (2)	0.0542 (7)	
H30	0.7878	−0.1722	1.0333	0.065*	
C31	0.9145 (3)	−0.08185 (11)	1.1101 (2)	0.0494 (7)	
H31	0.9591	−0.1072	1.1638	0.059*	
C32	0.9588 (3)	−0.03319 (11)	1.1318 (2)	0.0472 (6)	
H32A	1.0332	−0.0253	1.2004	0.057*	
C33	0.9364 (2)	0.05855 (10)	1.0712 (2)	0.0453 (6)	
H33	1.0090	0.0682	1.1394	0.054*	
C34	0.8710 (3)	0.09436 (10)	0.9907 (2)	0.0464 (6)	
H34	0.8970	0.1288	1.0034	0.056*	
C35	0.7639 (2)	0.07872 (9)	0.8881 (2)	0.0418 (6)	
H35	0.7204	0.1035	0.8330	0.050*	
C36	0.7998 (2)	−0.09593 (10)	1.0061 (2)	0.0411 (6)	
C37	0.7352 (2)	−0.05791 (9)	0.92362 (18)	0.0340 (5)	
C38	0.8945 (2)	0.00699 (9)	1.05175 (19)	0.0388 (6)	
C39	0.7839 (2)	−0.00576 (9)	0.94667 (18)	0.0326 (5)	
O8	0.33109 (16)	−0.06946 (6)	0.37794 (13)	0.0378 (4)	

### Atomic displacement parameters (Å^2^)

**Table:**

	*U*^11^	*U*^22^	*U*^33^	*U*^12^	*U*^13^	*U*^23^
Pr1	0.02559 (7)	0.03155 (7)	0.02071 (7)	0.00024 (5)	0.00814 (5)	−0.00049 (5)
O1	0.0421 (10)	0.0395 (9)	0.0467 (11)	0.0002 (8)	0.0233 (9)	−0.0037 (8)
O2	0.0559 (12)	0.0421 (10)	0.0618 (12)	−0.0025 (8)	0.0387 (10)	−0.0063 (8)
O3	0.0643 (13)	0.0493 (11)	0.0431 (11)	0.0116 (9)	0.0176 (10)	−0.0090 (8)
O4	0.0308 (9)	0.0521 (10)	0.0239 (9)	0.0076 (7)	0.0089 (7)	0.0022 (7)
O5	0.0315 (9)	0.0429 (9)	0.0259 (9)	0.0063 (7)	0.0067 (7)	0.0004 (7)
O6	0.0295 (9)	0.0438 (10)	0.0528 (11)	0.0070 (7)	0.0113 (8)	0.0006 (8)
O7	0.0473 (10)	0.0424 (9)	0.0284 (9)	−0.0123 (8)	0.0119 (8)	−0.0030 (7)
O9	0.0438 (11)	0.0493 (11)	0.0524 (12)	−0.0104 (8)	0.0133 (9)	−0.0103 (9)
N1	0.0341 (11)	0.0395 (11)	0.0248 (10)	0.0007 (9)	0.0106 (9)	−0.0006 (8)
N2	0.0317 (11)	0.0407 (11)	0.0277 (10)	−0.0013 (8)	0.0120 (9)	0.0011 (8)
C1	0.0314 (13)	0.0477 (15)	0.0273 (13)	0.0065 (11)	0.0086 (11)	−0.0031 (10)
C2	0.0475 (16)	0.0541 (16)	0.0436 (16)	0.0063 (13)	0.0223 (13)	−0.0095 (12)
C3	0.0484 (18)	0.097 (2)	0.056 (2)	0.0252 (17)	0.0181 (15)	−0.0155 (17)
C4	0.0482 (16)	0.0559 (16)	0.0384 (15)	0.0069 (13)	0.0235 (13)	−0.0087 (12)
C5	0.0551 (17)	0.0578 (18)	0.0479 (17)	−0.0002 (14)	0.0258 (15)	−0.0004 (13)
C6	0.062 (2)	0.086 (2)	0.053 (2)	0.0104 (17)	0.0297 (17)	0.0183 (17)
C7	0.0491 (19)	0.121 (3)	0.0392 (18)	0.001 (2)	0.0174 (15)	−0.0113 (19)
C8	0.056 (2)	0.085 (2)	0.067 (2)	−0.0097 (18)	0.0240 (18)	−0.0295 (19)
C9	0.061 (2)	0.0547 (17)	0.066 (2)	0.0027 (15)	0.0302 (17)	−0.0144 (15)
C10	0.0248 (12)	0.0290 (11)	0.0300 (13)	−0.0008 (9)	0.0096 (10)	0.0011 (9)
C11	0.0344 (13)	0.0442 (14)	0.0332 (13)	0.0043 (11)	0.0143 (11)	−0.0033 (10)
C12	0.0491 (17)	0.0701 (19)	0.0631 (19)	−0.0027 (14)	0.0376 (16)	0.0024 (15)
C13	0.0419 (15)	0.0383 (14)	0.0585 (18)	0.0066 (12)	0.0185 (14)	−0.0025 (12)
C14	0.056 (2)	0.0503 (18)	0.102 (3)	−0.0075 (15)	−0.0007 (19)	0.0150 (18)
C15	0.072 (3)	0.059 (2)	0.149 (4)	−0.0209 (19)	0.006 (3)	0.022 (2)
C16	0.095 (3)	0.052 (2)	0.130 (4)	−0.012 (2)	0.040 (3)	0.015 (2)
C17	0.098 (3)	0.059 (2)	0.088 (3)	0.007 (2)	0.031 (2)	0.0225 (19)
C18	0.061 (2)	0.0597 (19)	0.065 (2)	0.0054 (16)	0.0159 (17)	0.0046 (16)
C19	0.0309 (13)	0.0328 (12)	0.0331 (14)	−0.0004 (10)	0.0121 (11)	−0.0015 (10)
C20	0.0394 (14)	0.0412 (13)	0.0348 (14)	−0.0099 (11)	0.0113 (12)	−0.0003 (11)
C21	0.059 (2)	0.079 (2)	0.070 (2)	−0.0218 (16)	0.0396 (18)	−0.0074 (17)
C22	0.065 (2)	0.0302 (13)	0.0500 (17)	−0.0057 (12)	0.0255 (15)	−0.0036 (11)
C23	0.082 (2)	0.0430 (17)	0.066 (2)	−0.0033 (16)	0.0143 (19)	−0.0068 (15)
C24	0.159 (5)	0.066 (3)	0.063 (3)	0.002 (3)	0.028 (3)	−0.0196 (19)
C25	0.205 (6)	0.061 (2)	0.113 (4)	0.001 (3)	0.108 (4)	−0.018 (2)
C26	0.128 (4)	0.052 (2)	0.134 (4)	0.003 (2)	0.095 (3)	−0.003 (2)
C27	0.074 (2)	0.0387 (15)	0.077 (2)	−0.0040 (14)	0.0397 (19)	−0.0069 (14)
C28	0.0466 (16)	0.0465 (15)	0.0369 (14)	−0.0077 (12)	0.0139 (12)	0.0008 (11)
C29	0.066 (2)	0.0414 (15)	0.0558 (19)	−0.0033 (13)	0.0254 (16)	0.0071 (13)
C30	0.0571 (18)	0.0521 (16)	0.0478 (17)	0.0115 (14)	0.0223 (15)	0.0183 (13)
C31	0.0452 (16)	0.0628 (18)	0.0329 (15)	0.0164 (13)	0.0141 (12)	0.0130 (12)
C32	0.0366 (14)	0.0701 (19)	0.0235 (13)	0.0086 (13)	0.0066 (11)	0.0006 (12)
C33	0.0364 (14)	0.0622 (17)	0.0276 (13)	−0.0066 (12)	0.0089 (11)	−0.0138 (12)
C34	0.0459 (15)	0.0491 (15)	0.0372 (15)	−0.0103 (12)	0.0157 (13)	−0.0110 (12)
C35	0.0446 (15)	0.0412 (14)	0.0339 (14)	−0.0036 (11)	0.0155 (12)	−0.0022 (11)
C36	0.0395 (14)	0.0491 (15)	0.0355 (14)	0.0109 (11)	0.0195 (12)	0.0100 (11)
C37	0.0320 (13)	0.0440 (13)	0.0289 (13)	0.0056 (10)	0.0175 (11)	0.0031 (10)
C38	0.0310 (13)	0.0581 (16)	0.0254 (12)	0.0024 (11)	0.0129 (10)	−0.0037 (11)
C39	0.0280 (12)	0.0461 (13)	0.0242 (11)	0.0032 (10)	0.0136 (10)	−0.0012 (10)
O8	0.0433 (10)	0.0369 (9)	0.0294 (9)	−0.0072 (7)	0.0153 (8)	−0.0026 (7)

### Geometric parameters (Å, °)

**Table:**

Pr1—O5^i^	2.4215 (15)	C13—C14	1.372 (4)
Pr1—O7	2.4320 (15)	C14—C15	1.382 (5)
Pr1—O8^i^	2.4657 (15)	C14—H14	0.9300
Pr1—O2	2.5117 (17)	C15—C16	1.351 (5)
Pr1—O1	2.5324 (16)	C15—H15	0.9300
Pr1—O4	2.5501 (15)	C16—C17	1.374 (5)
Pr1—N1	2.6199 (18)	C16—H16	0.9300
Pr1—O5	2.6755 (15)	C17—C18	1.376 (5)
Pr1—N2	2.6782 (18)	C17—H17	0.9300
Pr1—Pr1^i^	4.0785 (2)	C18—H18	0.9300
O1—C1	1.249 (3)	C19—O8	1.247 (3)
O2—C1	1.253 (3)	C19—C20	1.529 (3)
O3—C4	1.377 (3)	C20—C21	1.510 (4)
O3—C2	1.417 (3)	C20—H20	0.9800
O4—C10	1.240 (3)	C21—H21A	0.9600
O5—C10	1.266 (3)	C21—H21B	0.9600
O5—Pr1^i^	2.4215 (15)	C21—H21C	0.9600
O6—C13	1.368 (3)	C22—C27	1.369 (4)
O6—C11	1.425 (3)	C22—C23	1.381 (4)
O7—C19	1.257 (3)	C23—C24	1.373 (6)
O9—C22	1.371 (3)	C23—H23	0.9300
O9—C20	1.413 (3)	C24—C25	1.358 (6)
N1—C35	1.327 (3)	C24—H24	0.9300
N1—C39	1.359 (3)	C25—C26	1.357 (6)
N2—C28	1.317 (3)	C25—H25	0.9300
N2—C37	1.363 (3)	C26—C27	1.374 (4)
C1—C2	1.534 (3)	C26—H26	0.9300
C2—C3	1.515 (4)	C27—H27	0.9300
C2—H2	0.9800	C28—C29	1.393 (4)
C3—H3A	0.9600	C28—H28	0.9300
C3—H3B	0.9600	C29—C30	1.356 (4)
C3—H3C	0.9600	C29—H29	0.9300
C4—C9	1.376 (4)	C30—C36	1.395 (4)
C4—C5	1.380 (4)	C30—H30	0.9300
C5—C6	1.386 (4)	C31—C32	1.335 (4)
C5—H5	0.9300	C31—C36	1.432 (4)
C6—C7	1.371 (5)	C31—H31	0.9300
C6—H6	0.9300	C32—C38	1.429 (3)
C7—C8	1.374 (5)	C32—H32A	0.9300
C7—H7	0.9300	C33—C34	1.355 (4)
C8—C9	1.364 (4)	C33—C38	1.398 (3)
C8—H8	0.9300	C33—H33	0.9300
C9—H9	0.9300	C34—C35	1.397 (3)
C10—C11	1.519 (3)	C34—H34	0.9300
C11—C12	1.510 (3)	C35—H35	0.9300
C11—H11	0.9800	C36—C37	1.407 (3)
C12—H12A	0.9600	C37—C39	1.435 (3)
C12—H12B	0.9600	C38—C39	1.414 (3)
C12—H12C	0.9600	O8—Pr1^i^	2.4657 (15)
C13—C18	1.368 (4)		
			
O5^i^—Pr1—O7	73.34 (5)	C9—C8—C7	120.5 (3)
O5^i^—Pr1—O8^i^	77.97 (5)	C9—C8—H8	119.8
O7—Pr1—O8^i^	134.03 (5)	C7—C8—H8	119.8
O5^i^—Pr1—O2	87.11 (6)	C8—C9—C4	120.4 (3)
O7—Pr1—O2	85.58 (6)	C8—C9—H9	119.8
O8^i^—Pr1—O2	128.21 (5)	C4—C9—H9	119.8
O5^i^—Pr1—O1	77.06 (5)	O4—C10—O5	122.5 (2)
O7—Pr1—O1	128.56 (6)	O4—C10—C11	120.57 (19)
O8^i^—Pr1—O1	76.72 (5)	O5—C10—C11	116.88 (19)
O2—Pr1—O1	51.55 (5)	O4—C10—Pr1	58.35 (11)
O5^i^—Pr1—O4	123.14 (5)	O5—C10—Pr1	64.18 (11)
O7—Pr1—O4	89.95 (5)	C11—C10—Pr1	178.35 (16)
O8^i^—Pr1—O4	76.57 (5)	O6—C11—C12	106.9 (2)
O2—Pr1—O4	146.62 (6)	O6—C11—C10	111.47 (19)
O1—Pr1—O4	141.43 (5)	C12—C11—C10	110.0 (2)
O5^i^—Pr1—N1	145.74 (6)	O6—C11—H11	109.5
O7—Pr1—N1	139.03 (6)	C12—C11—H11	109.5
O8^i^—Pr1—N1	80.60 (5)	C10—C11—H11	109.5
O2—Pr1—N1	85.49 (6)	C11—C12—H12A	109.5
O1—Pr1—N1	72.18 (6)	C11—C12—H12B	109.5
O4—Pr1—N1	76.43 (5)	H12A—C12—H12B	109.5
O5^i^—Pr1—O5	73.81 (5)	C11—C12—H12C	109.5
O7—Pr1—O5	69.40 (5)	H12A—C12—H12C	109.5
O8^i^—Pr1—O5	68.52 (5)	H12B—C12—H12C	109.5
O2—Pr1—O5	151.85 (6)	C18—C13—O6	116.3 (2)
O1—Pr1—O5	138.28 (5)	C18—C13—C14	119.2 (3)
O4—Pr1—O5	49.65 (5)	O6—C13—C14	124.4 (3)
N1—Pr1—O5	121.82 (5)	C13—C14—C15	119.4 (3)
O5^i^—Pr1—N2	148.38 (6)	C13—C14—H14	120.3
O7—Pr1—N2	77.14 (5)	C15—C14—H14	120.3
O8^i^—Pr1—N2	132.35 (5)	C16—C15—C14	121.7 (4)
O2—Pr1—N2	79.51 (6)	C16—C15—H15	119.2
O1—Pr1—N2	114.34 (5)	C14—C15—H15	119.2
O4—Pr1—N2	67.27 (5)	C15—C16—C17	118.9 (3)
N1—Pr1—N2	61.92 (6)	C15—C16—H16	120.6
O5—Pr1—N2	106.09 (5)	C17—C16—H16	120.6
O5^i^—Pr1—C1	83.26 (6)	C16—C17—C18	120.1 (3)
O7—Pr1—C1	108.79 (7)	C16—C17—H17	119.9
O8^i^—Pr1—C1	102.53 (6)	C18—C17—H17	119.9
O2—Pr1—C1	25.89 (6)	C13—C18—C17	120.7 (3)
O1—Pr1—C1	25.82 (6)	C13—C18—H18	119.7
O4—Pr1—C1	151.81 (6)	C17—C18—H18	119.7
N1—Pr1—C1	75.66 (6)	O8—C19—O7	126.9 (2)
O5—Pr1—C1	156.59 (6)	O8—C19—C20	119.4 (2)
N2—Pr1—C1	95.86 (6)	O7—C19—C20	113.7 (2)
O5^i^—Pr1—C10	98.88 (6)	O9—C20—C21	107.2 (2)
O7—Pr1—C10	78.82 (6)	O9—C20—C19	115.1 (2)
O8^i^—Pr1—C10	70.96 (5)	C21—C20—C19	110.1 (2)
O2—Pr1—C10	160.83 (6)	O9—C20—H20	108.1
O1—Pr1—C10	147.52 (6)	C21—C20—H20	108.1
O4—Pr1—C10	24.45 (5)	C19—C20—H20	108.1
N1—Pr1—C10	98.99 (6)	C20—C21—H21A	109.5
O5—Pr1—C10	25.20 (5)	C20—C21—H21B	109.5
N2—Pr1—C10	86.16 (6)	H21A—C21—H21B	109.5
C1—Pr1—C10	172.38 (7)	C20—C21—H21C	109.5
O5^i^—Pr1—Pr1^i^	39.05 (4)	H21A—C21—H21C	109.5
O7—Pr1—Pr1^i^	66.36 (4)	H21B—C21—H21C	109.5
O8^i^—Pr1—Pr1^i^	68.66 (4)	C27—C22—O9	125.9 (2)
O2—Pr1—Pr1^i^	123.26 (4)	C27—C22—C23	120.3 (3)
O1—Pr1—Pr1^i^	110.90 (4)	O9—C22—C23	113.7 (3)
O4—Pr1—Pr1^i^	84.26 (3)	C24—C23—C22	119.0 (4)
N1—Pr1—Pr1^i^	146.78 (4)	C24—C23—H23	120.5
O5—Pr1—Pr1^i^	34.76 (3)	C22—C23—H23	120.5
N2—Pr1—Pr1^i^	133.43 (4)	C25—C24—C23	120.8 (4)
C1—Pr1—Pr1^i^	122.16 (5)	C25—C24—H24	119.6
C10—Pr1—Pr1^i^	59.87 (4)	C23—C24—H24	119.6
C1—O1—Pr1	92.14 (14)	C26—C25—C24	119.9 (4)
C1—O2—Pr1	93.02 (15)	C26—C25—H25	120.1
C4—O3—C2	119.1 (2)	C24—C25—H25	120.1
C10—O4—Pr1	97.21 (13)	C25—C26—C27	120.8 (4)
C10—O5—Pr1^i^	162.15 (15)	C25—C26—H26	119.6
C10—O5—Pr1	90.62 (13)	C27—C26—H26	119.6
Pr1^i^—O5—Pr1	106.19 (5)	C22—C27—C26	119.3 (3)
C13—O6—C11	117.61 (19)	C22—C27—H27	120.4
C19—O7—Pr1	140.05 (15)	C26—C27—H27	120.4
C22—O9—C20	119.6 (2)	N2—C28—C29	123.8 (2)
C35—N1—C39	118.2 (2)	N2—C28—H28	118.1
C35—N1—Pr1	120.41 (15)	C29—C28—H28	118.1
C39—N1—Pr1	120.78 (14)	C30—C29—C28	118.9 (3)
C28—N2—C37	117.5 (2)	C30—C29—H29	120.6
C28—N2—Pr1	122.93 (16)	C28—C29—H29	120.6
C37—N2—Pr1	118.46 (14)	C29—C30—C36	119.9 (2)
O1—C1—O2	122.5 (2)	C29—C30—H30	120.0
O1—C1—C2	119.3 (2)	C36—C30—H30	120.0
O2—C1—C2	118.1 (2)	C32—C31—C36	121.4 (2)
O1—C1—Pr1	62.04 (12)	C32—C31—H31	119.3
O2—C1—Pr1	61.09 (12)	C36—C31—H31	119.3
C2—C1—Pr1	173.89 (17)	C31—C32—C38	121.4 (2)
O3—C2—C3	106.5 (2)	C31—C32—H32A	119.3
O3—C2—C1	111.9 (2)	C38—C32—H32A	119.3
C3—C2—C1	109.7 (2)	C34—C33—C38	119.9 (2)
O3—C2—H2	109.5	C34—C33—H33	120.0
C3—C2—H2	109.5	C38—C33—H33	120.0
C1—C2—H2	109.5	C33—C34—C35	119.0 (2)
C2—C3—H3A	109.5	C33—C34—H34	120.5
C2—C3—H3B	109.5	C35—C34—H34	120.5
H3A—C3—H3B	109.5	N1—C35—C34	123.2 (2)
C2—C3—H3C	109.5	N1—C35—H35	118.4
H3A—C3—H3C	109.5	C34—C35—H35	118.4
H3B—C3—H3C	109.5	C30—C36—C37	117.4 (2)
C9—C4—O3	115.2 (3)	C30—C36—C31	123.4 (2)
C9—C4—C5	120.1 (3)	C37—C36—C31	119.2 (2)
O3—C4—C5	124.7 (2)	N2—C37—C36	122.4 (2)
C4—C5—C6	118.6 (3)	N2—C37—C39	118.3 (2)
C4—C5—H5	120.7	C36—C37—C39	119.3 (2)
C6—C5—H5	120.7	C33—C38—C39	117.8 (2)
C7—C6—C5	121.2 (3)	C33—C38—C32	123.4 (2)
C7—C6—H6	119.4	C39—C38—C32	118.8 (2)
C5—C6—H6	119.4	N1—C39—C38	121.8 (2)
C6—C7—C8	119.2 (3)	N1—C39—C37	118.3 (2)
C6—C7—H7	120.4	C38—C39—C37	119.9 (2)
C8—C7—H7	120.4	C19—O8—Pr1^i^	134.83 (14)

Symmetry codes: (i) −*x*+1, −*y*, −*z*+1.
